# Frutescone O from *Baeckea frutescens* Blocked TLR4-Mediated Myd88/NF-κB and MAPK Signaling Pathways in LPS Induced RAW264.7 Macrophages

**DOI:** 10.3389/fphar.2021.643188

**Published:** 2021-04-27

**Authors:** Xiaobing Lin, Junhan Zhang, Decai Fan, Jiqin Hou, Hao Wang, Lin Zhu, Ruina Tian, Xiaofei An, Ming Yan

**Affiliations:** ^1^ Institute of Pharmaceutical Science, China Pharmaceutical University, Nanjing, China; ^2^ Department of Natural Medicinal Chemistry, China Pharmaceutical University, Nanjing, China; ^3^ Department of Endocrinology, Affiliated Hospital of Nanjing University of Chinese Medicine, Nanjing, China

**Keywords:** Frutescone O, MyD88, NF-κB, MAPKs, TLR4 (toll-like receptor 4)

## Abstract

Frutescone O was isolated from the aerial parts of *Baeckea frutescens* L., which was commonly used as a folk medicinal material for treating anti-inflammatory disease in South East Asia. This study aimed to investigate the anti-inflammatory activity and related signaling cascade of Frutescone O (Fru) in LPS induced RAW264.7 cells. The anti-inflammation activity of Frutescone O was determined according to the inhibitory effects on the secretion of nitric oxide (NO), expression of inducible NO synthase, and pro-inflammatory cytokines. The regulation of Myeloid differentiation factor 88 (Myd88), inhibition of NF-κB, and MAPK pathways were further investigated for molecular mechanisms. Fru significantly decreased the expression of iNOS and the production of NO in LPS-stimulated RAW264.7 cells. It also dose-dependently suppressed LPS induced expression of IL-1β, IL-6, and TNF-α. Furthermore, Fru remarkably inhibited the upregulation of NF-κB (p50) expression in the nucleus and the phosphorylation ratio of p38, JNK, ERK, and Myd88 signaling protein. The molecular docking and cellular thermal shift assay (CETSA) results indicated that Fru participated in a robust and stable interaction with the active site of TLR4-MD2. Thus, Fru suppressed the LPS induced inflammation in RAW264.7 cells by blocking the TLR4 mediated signal transduction through the NF-κB and MAPK signaling pathways and inhibiting the Myd88 and iNOS expression.

## Introduction

*Baeckea frutescens* L. is a small tree found in mountainous regions. It is a medicinal plant of the family Myrtaceae and subfamily Myrtoideae. As a traditional folk medicine in South East Asia, it is often used to treat inflammatory diseases, including rheumatism, snake bites, dermatitis, and the common cold (Hou et al., 2017). Fru is a meroterpenoid isolated from *Baeckea frutescens* and has various medical functions (Razmavar et al., 2014). Fru was supposed to be related to the NF-κB signaling pathway regulation *via* the suppression of p65 nuclear translocation in LPS-induced macrophages (Hou et al., 2017). In the present study, the anti-inflammatory mechanism of Fru is further studied in LPS-stimulated RAW264.7 cells.

Inflammation is a common and necessary pathophysiological process. It acts as a self-defense response towards invading pathogens, damaged cells, and other endogenous or exogenous irritants (Chen et al., 2015). However, an excessive inflammatory response can lead to various inflammation-related diseases such as sepsis, osteoarthritis, and rheumatoid arthritis (Haworth and Levy, 2007). The gastrointestinal and cardiovascular side effects of nonsteroidal anti-inflammatory drugs mean that it is necessary to develop new candidates (Nussmeier et al., 2005; Solomon et al., 2005). Non-steroidal anti-inflammatory drugs (NSAIDs) are widely used acutely as analgesics and chronically as analgesics to reduce pain and inflammation in many patients with arthritic conditions. Both the therapeutic and adverse effects of NSAIDs are due to inhibition of the cyclooxygenase (COX) enzyme, present in two isoforms, the constitutive COX-1, and the inducible COX-2. Presently, the major side effects of NSAIDs are gastrointestinal complications and COX-2 selective NSAIDs, designed to prevent gastrointestinal toxicity, which seem to be predisposed to an increased cardiovascular risk. (Harirforoosh et al., 2013). Consequently, there is a need to develop new anti-inflammatory drugs.

Macrophages are a necessary component of the immunological system and have a stimulation effect on inflammation. Activated macrophages produce a wide variety of inflammatory mediators, including nitric oxide (NO) and prostaglandin E_2_ (PGE_2_), as well as numerous cytokines, such as tissue necrosis factor-α (TNF-α), interleukin-1β (IL-1β), and interleukin-1 (IL-6) (Aderem and Ulevitch, 2000). Lipopolysaccharide (LPS) is recognized as a toll-like receptor 4 (TLR4) and can activate macrophages through the NF-κB pathway and MAPKs pathways (Kawai and Akira, 2010).

Nuclear transcription factor-κB (NF-κB) is a common transcription factor regulating pro-inflammatory mediators and cytokines (Schmid and Birbach, 2008). The activated NF-κB translocates into the nucleus and promotes the expression of various proinflammatory cytokines, such as NO, PGE_2_, TNF-α (Beg et al., 1992; Lee et al., 1998; Pennington et al., 2001; Li and Verma, 2002; Tian et al., 2005). Moreover, MAPKs are signaling molecules that regulate the cellular response to cytokines, stress, and inflammation (Swaroop et al., 2016). The activation of the NF-κB signaling pathway and MAPKs signaling pathway is dependent on the activation of toll-like receptors (TLRs) (Kawai and Akira, 2010).

## Materials and Methods

### Chemicals and Reagents

Dimethyl sulfoxide (DMSO) and Lipopolysaccharides (LPS) were purchased from Sigma Chemical Co. (United States). Nitric Oxide (NO) Griess Reagent was from Beyotime Institute of Biotechnology (Haimen, China). The nuclear protein extraction kit was from Vazyme (Nanjing, China). Ginsenoside Rb1 (Cat# HY-N0039) was purchased from MedChemExpress (Shanghai, China). All of the antibodies were purchased from Cell Signaling TechnologyInc (United States).

### Extraction and Isolation of Fru

**FIGURE 1:**
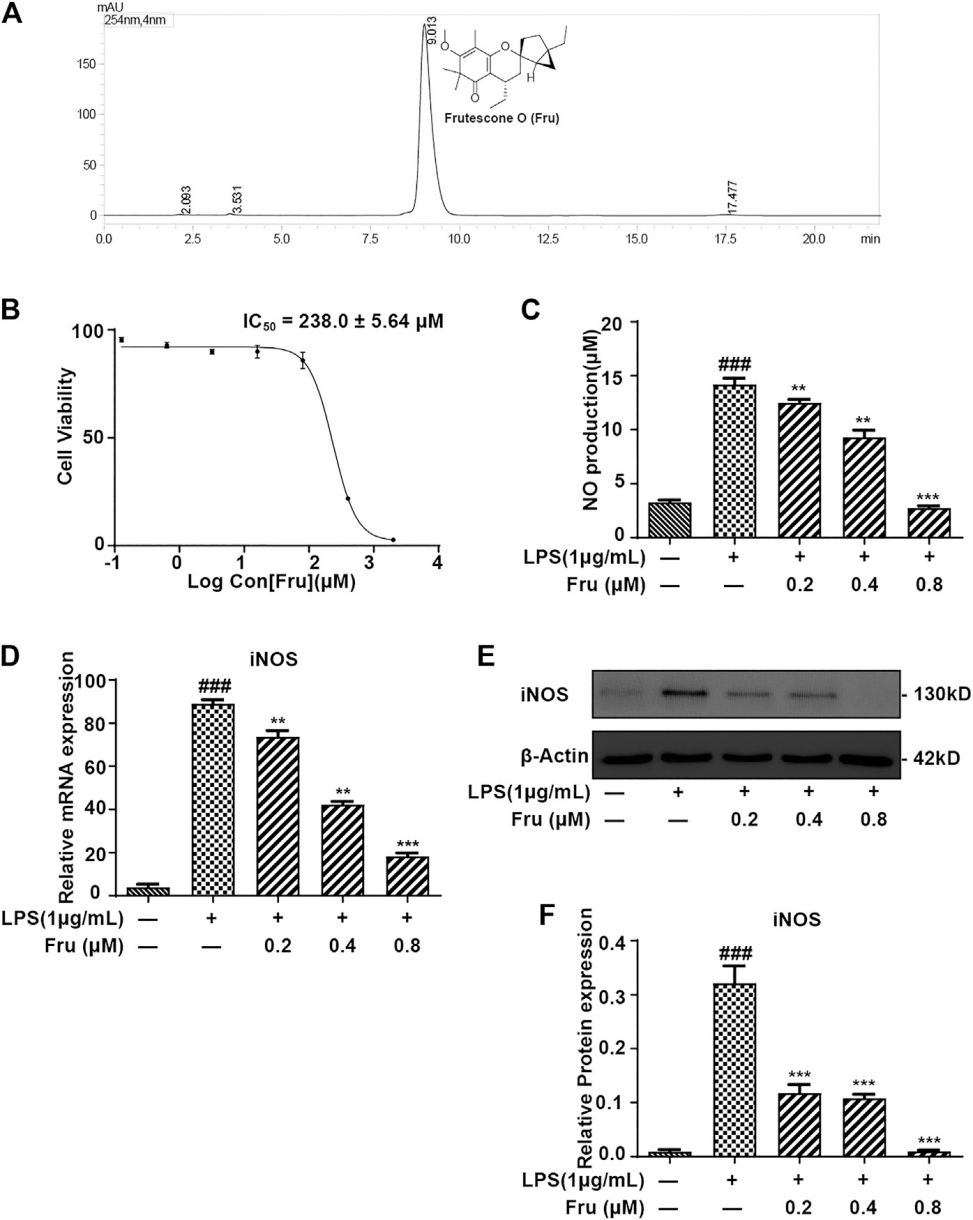
Fru suppressed the production of NO in LPS induced RAW264.7 cells. **(A)** Structure and HPLC chromatogram of Fru isolated from *Baeckea frutescens* L. The purity of Fru was determined to be over 98% by normalization of the peak area detected by HPLC-UV. **(B)** Effect of Fru on cell viability of RAW264.7 cells. Cells were incubated with Fru (0.1–2000 μM) for 24 h. Cell viability was evaluated by the Alamar Blue assay. **(C)** Fru suppressed the production of NO in LPS-induced RAW264.7 cells. Cells were incubated with different concentrations of Fru (0.2, 0.4, and 0.8 μM) and 1 μg/ml LPS for 18 h. NO production in the cell culture supernatant was detected by the Griess assay. **(D)** Effect of Fru on iNOS mRNA expression. Relative mRNA expression levels of iNOS were measured with qRT-PCR and normalized to GAPDH. **(E, F)** Effects of Fru on iNOS protein expression. The expression of iNOS was determined with Western Blot analysis. β-actin was used as the internal control. Quantitative data was represented as mean ± SD (*n* = 3, ^###^ p < 0.001 vs. Control; **p < 0.01, ***p < 0.001 vs. LPS).

Fru was extracted and isolated from the petroleum ether fraction of the aerial parts of *Baeckea frutescens* as described previously (Hou et al., 2017). Chromatographic analysis was performed on a Shimadzu VP-ODS (150 × 4.6 mm, 5 μm), using methanol: water (90:10, v/v) as the mobile phase at a flow rate of 1 ml/min at 30°C. The purity of compound Fru was determined to be over 98% by normalization of the peak area detected by HPLC-UV (Figure 1A).

### Cell Culture

The RAW264.7 cell line was obtained from the American Type Culture Collection (United States), which was cultured in Dulbecco’s modified Eagle’s medium (Gibco, United States), 10% fetal bovine serum (Gibco, United States), 100 U/ml penicillin, and 100 μg/ml streptomycin (Sigma, United States) in the atmosphere of 37°C and humidified 5% CO_2_.

### Cell Viability Assay

RAW264.7 cells (1×10^6^ cells/mL) were cultured for 12 h. Different concentrations of Fru (0.13, 0.64, 3.2, 16, 80, 400, 2000 μM) were added into the 384-well microplate and incubated for 16 h. Then, Alamar Blue (Sigma, United States), at the concentration of 10% (v/v) was added into 384-well microplates and incubated for another 8 h. Fluorescence was detected (wavelength: 560/600 nm) through a Safire2™ microplate reader (Tecan, Switzerland). The method of cell viability detection is described as previous (Chen et al., 2015; Yu et al., 2017).

### NO Production Determination

RAW264.7 cells (1×10^6^ cells/ml) were cultured with various concentrations of Fru (0.2, 0.4, and 0.8 μM) in 96-well microplates for 2 h and incubated with 1 μg/ml LPS for 18 h. The NO concentration in the supernatant was determined by the Griess assay. Briefly, 50 μl of supernatant was added in 96-well microplates, followed by Griess reagent I (50 μl) and Griess reagent II (50 μl), and the absorbance (wavelength: 540 nm) was detected through a Safire2™ microplate reader (Tecan, Switzerland).

### Real-Time Polymerase Chain Reaction (RT-PCR)

RAW264.7 cells (1×10^6^ cells/ml) were treated with various concentrations of Fru (0.2, 0.4, and 0.8 μM) for 2 h in 6-well microplates and stimulated with LPS (1 μg/ml) for 18 h. The total RNA was extracted by Total RNA Extraction Reagent (Vazyme, China) according to the manufacturer’s specification. The concentration of RNA was evaluated by spectrophotometric analysis with Gene Quant Pro spectrophotometer (Amersham Biosciences, United States). RNA (1 μg) was reverse-transcribed to cDNA by HiScript™ Q-RT Super Mix (Fcmacs, China). qRT-PCR was displayed by SYBRs Green Master Mix (Fcmacs, China) on an iCycleriQ™ five Multicolor Real-Time PCR Detection System (Bio-Rad, United States). The sequences of primers are shown in (Table 1).

**TABLE 1:** Specific primers for RT-PCR.

Gene	Primer
iNOS	Forward: 5′-TTG​GGT​CTT​GTT​CAC​TCC​ACG-3′
	Reverse: 5′-GGC​TGA​GAA​CAG​CAC​AAG​GG-3′
TNF-α	Forward: 5′-GTA​GCC​CAC​GTC​GTA​GCA​A-3′
	Reverse: 5′-GTG​AGG​AGC​ACG​TAG​TCG​G-3′
IL-6	Forward: 5′-CGG​CCT​TCC​CTA​CTT​CAC​AA-3′
	Reverse: 5′-TCT​GCA​AGT​GCA​TCA​TCG​TT-3′
IL-1β	Forward: 5′-GGC​TGT​GGA​GAA​GCT​GTG​GC-3′
	Reverse: 5′-GGG​TGG​GTG​TGC​CGT​CTT​TC-3′
Myd88	Forward: 5′-ATC​GCT​GTT​CTT​GAA​CCC​TCG-3′
	Reverse: 5′-CTC​ACG​GTC​TAA​CAA​GGC​CAG-3′
GAPDH	Forward: 5′-CTA​GGA​CTG​GAT​AAG​CAG​GGC-3′
	Reverse: 5′-ATC​CGT​TCA​CAC​CGA​CCT​TC-3′

### Cytoplasmic and Nuclear Extraction

RAW264.7 cells (1×10^6^ cells/ml) were cultured with various concentrations of Fru (0.2, 0.4, and 0.8 μM) in a 6-well microplate for 2 h and stimulated with or without 1 μg/ml LPS for different times. The stimulation period was 18 h for iNOS; 6 h for *p*-ERK1/2, ERK1/2, *p*-JNK, JNK, p-p38 MAPK, p38 MAPK; 30 min for IκBα, p- IκBα; 1 h for p50, and 8 h for Myd88. Meanwhile, the nuclear protein (p50) was extracted by Nuclear Protein Extraction Kit (Thermo, United States) according to the manufacture’s specifications.

### Western Blot Analysis

BCA assay kit (Beyotime, China) was used to measure the concentration of protein samples. The protein sample (20 μg) was separated by sodium dodecyl sulfate-polyacrylamide gel electrophoresis before being electrically transferred onto PVDF membranes. After blocking with 30% bovine serum albumin (BSA) for 1 h at room temperature, the PVDF membranes were incubated with primary antibodies at 4°C overnight and incubated with HRP-conjugated secondary antibodies for 1 h at room temperature. ECL reagents (Vazyme, China) were used to stimulate chemiluminescent signals, which were measured and evaluated by the ChemiDoc XRS imaging system (Bio-Rad, United States).

### Computer Simulation of Molecular Docking

The chemical structure of the test was sketched in ChemDraw 19.0, and save in Mol2 format. X-ray crystal structure of TLR4- myeloid differential protein-2 (MD2) complex (PDB ID: 3fxi, resolution = 3.10 Å) were obtained from the Protein Data Bank (PDB) (http://www1.rcsb.org). The protein required in docking was prepared by Discovery Studio 4.5 (DS 4.5) software. Ligands and water molecules were removed from the crystal structures of the protein, and hydrogen atoms were added. This docking took a conformation of a ligand, a target protein structure, a sphere defining the active site of the protein based on the co-crystallized ligand into DS 4.5. The protein structure was treated as rigid, and the comforms of ligand as flexible during the docking process. CDOCKER, a docking module, was used to simulate the binding style between protein complex and compound. All the parameters were set as the default mode. A 3D diagram of their interaction was created to confirm the results, and their docking pose was presented for the analysis of the interactions, including hydrogen bond, hydrophobic bond, π-π interaction, and so on. The corresponding results were evaluated based upon the-CDOCKER interaction energy, hydrogen bond interaction, and the binding mode pattern.

### Cellular Thermal Shift Assay

As previously described, RAW264.7 cells were seeded into T75 cell culture flasks (2×10^6^ cells/ml) and treated by serum-free medium, Fru (0.8 μM), and Rb1 (40 μM) for 12 h, respectively, (Gao et al., 2020). The cells were homogenized by a grinder (20 strokes) before being centrifuged at 4°C 10,000 × g for 10 min. The supernatant was collected and quantified with a BCA assay. The protein of each group was equally divided into eight tubes (100 μl/tube), and heated at 37, 40, 44, 48, 52, 56, 60 and 64°C for 5 min, respectively. Then, all samples were centrifuged at 4°C 10,000 ×g for 20 min and the supernatants were collected for western blot analysis.

### Statistical Analysis

Statistical analysis was performed in GraphPad Prism 6.0 software (GraphPad, United States). Results were performed as *mean ± SD* from three independent experiments. Data were compared by one-way ANOVA followed by Dunnett’s Multiple Comparison Test. The differences were considered statistically significant when *p < 0.05*. The replications are *n* = 3 for the various treatment conditions in each experiment for statistical analysis and the IC_50_s were calculated by nonlinear regression.

## Results

### Structure and HPLC Chromatogram of Fru Isolated From *Baeckea frutescens* L

The purity of Fru was determined to be over 98% by normalization of the peak area detected by HPLC-UV (Figure 1A).

### Anti-Inflammatory Effect of Fru in LPS Stimulated RAW264.7 Cells

The cytotoxicity results showed that the IC_50_ of Fru was 238.0 ± 5.64 μM on RAW264.7 cells (Figure 1B). In the Griess assay, Fru significantly blocked the LPS-induced production of NO in a dose-dependent manner (Figure 1C). The relative inhibition percentage of Fru (0.2, 0.4 and 0.8 μM) was 4.87 ± 1.91%, 55.04 ± 6.16%, and 84.42 ± 2.78%, respectively. There was a significant difference between the value of NO production in the absence and presence of LPS (3.27 ± 0.18 μM *vs* 14.21 ± 0.46 μM). The dose-dependent inhibition of NO production in Fru (0.2, 0.4 and 0.8 μM) was 12.50 ± 0.30 μM, 9.29 ± 0.67 μM, and 2.73 ± 0.21 μM, respectively. Meanwhile, Fru dose-dependently decreased the mRNA (73.75 ± 2.75, 42.24 ± 1.51, 18.41 ± 1.43 *vs* 88.99 ± 1.86, respectively) and protein (0.12 ± 0.02, 0.11 ± 0.01, 0.01 ± 0.00 *vs* 0.32 ± 0.03, respectively) expression of iNOS in LPS-stimulated RAW264.7 cells (Figures 1D–F).

### Fru Inhibited the Transcription of Proinflammatory Cytokines

**FIGURE 2:**
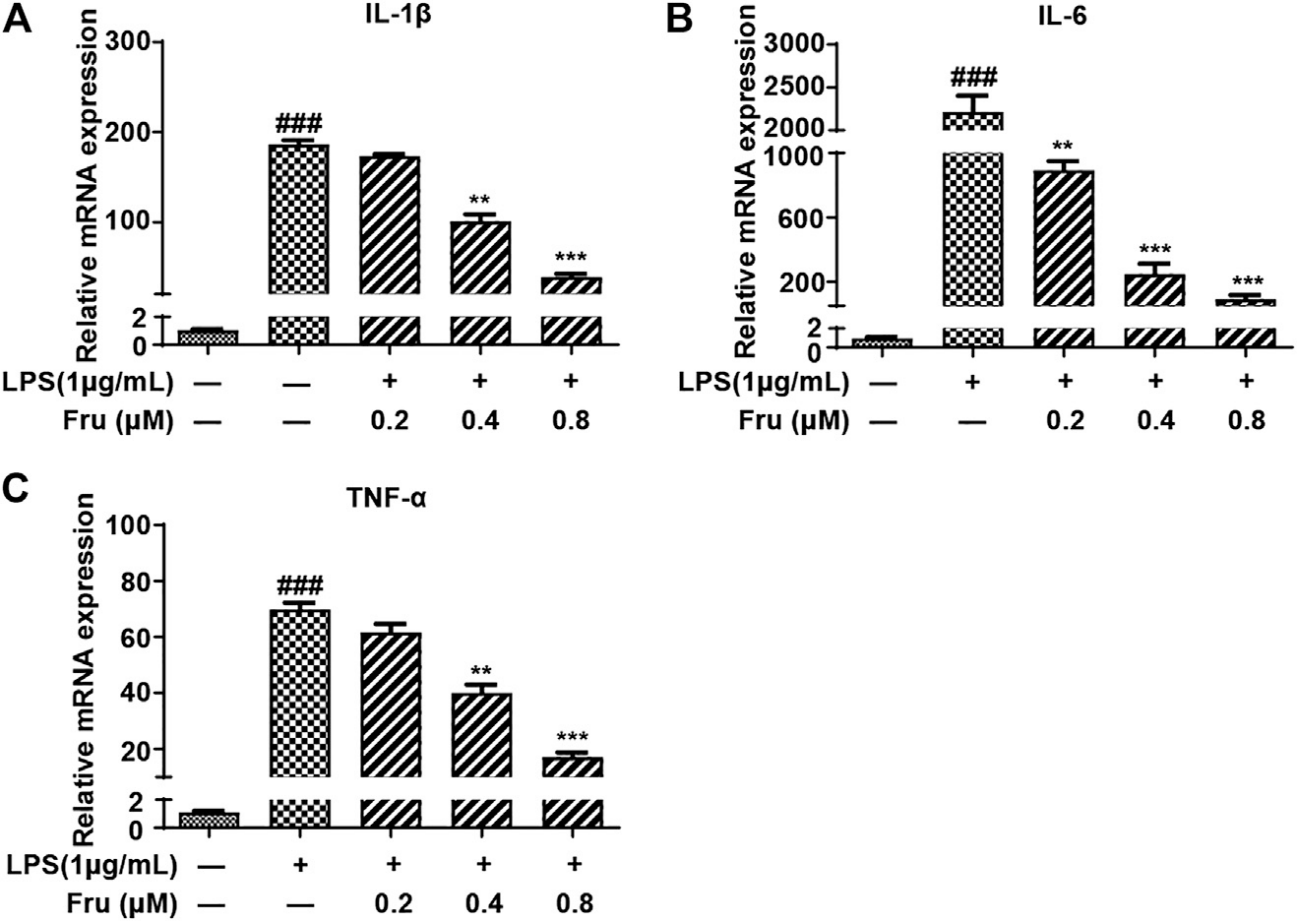
Fru suppressed mRNA expression of proinflammatory cytokines in LPS-induced RAW264.7 cells. Cells were treated with different concentrations of Fru (0.2, 0.4, and 0.8 μM) for 2 h and then stimulated with or without LPS (1 μg/ml) for 18 h. The relative mRNA expression levels of IL-1β (Panel **(A)**), IL-6 (Panel **(B)**), and TNF-α (Panel **(C)**) were measured with qRT-PCR and normalized to GAPDH. Quantitative data was represented as mean ± SD (*n* = 3, ^###^ *p* < 0.001 vs. Control; **p < 0.01, ***p < 0.001 vs. LPS).

The inhibitory effects of Fru on LPS-induced expression of proinflammatory cytokines were determined using qRT-PCR analysis. Fru dose-dependently suppressed LPS induced expression of IL-1β (173.30 ± 2.17, 101.32 ± 7.54, 39.10 ± 3.35 *vs* 186.74 ± 4.42, respectively), IL-6 (891.71 ± 58.52, 248.83 ± 62.87, 89.84 ± 27.57 *vs* 2,217.21 ± 188.32, respectively), and TNF-α (61.67 ± 3.07, 40.16 ± 2.86, 17.17 ± 1.58 *vs* 69.87 ± 2.45, respectively) (Figure 2).

### Effects of Fru on the NF-κB Signaling Pathway in LPS-Stimulated RAW264.7 Cells

**FIGURE 3:**
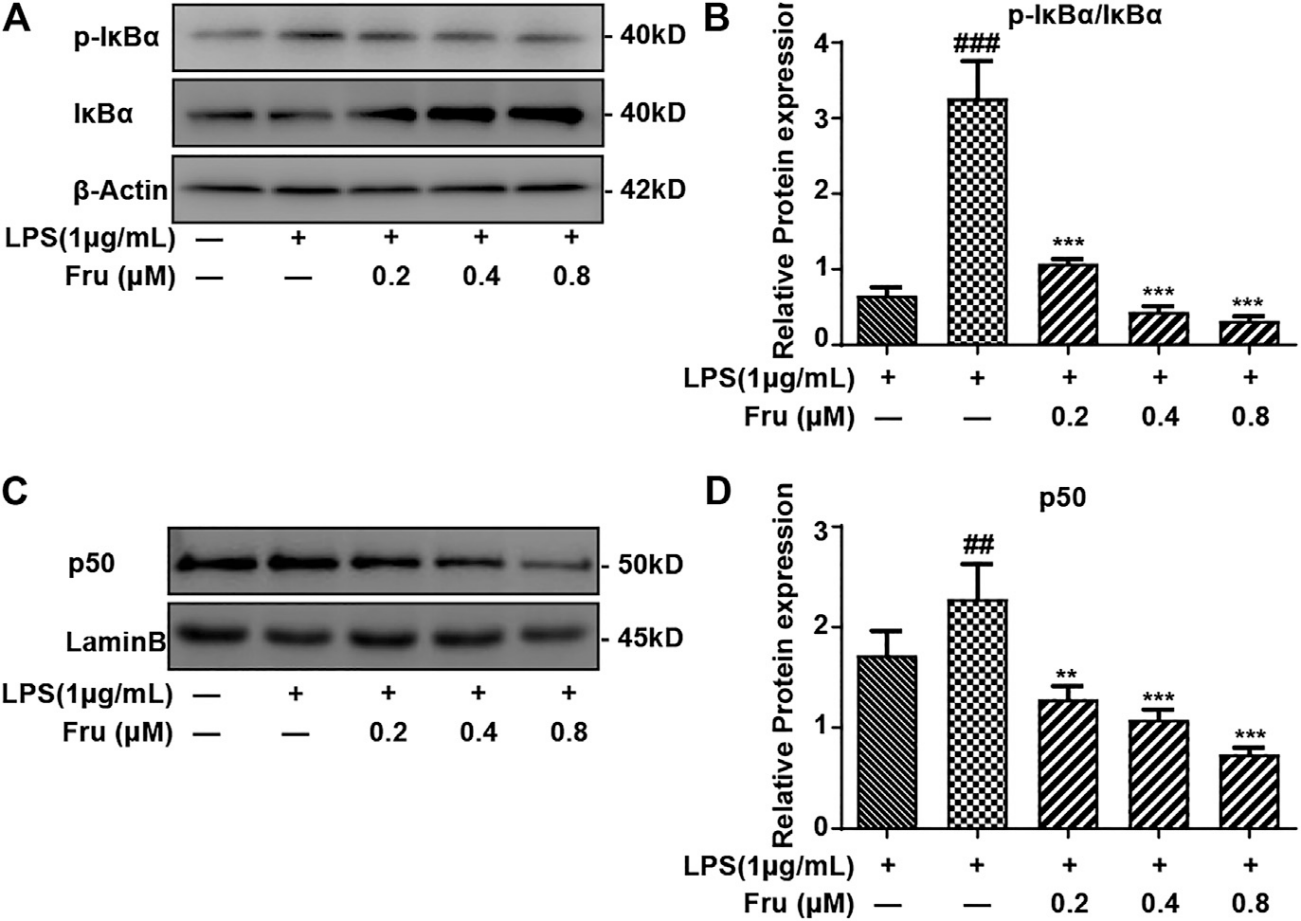
Effect of Fru on activation of the NF-κB pathway in LPS-stimulated RAW264.7 cells. RAW264.7 cells were incubated with different concentrations of Fru (0.2, 0.4, and 0.8 μM) for 2 h before treated with 1 μg/ml LPS for different times (30 min for IκBα, p- IκBα; and 1 h for p50). The protein level of p- IκBα/IκBα (Panel **(A,B)**), and p50 (Panel **(C,D)**) were analyzed by Western Blot. Lamin B and β-actin were used as the internal control. Quantitative data were represented as mean ± SD (*n* = 3, ^##^ *p* < 0.01, ^###^ *p* < 0.001 vs. Control; **p < 0.01, ***p < 0.001 vs. LPS).

It was indicated that the expression of *p*-IκBα/IκBα protein (1.08 ± 0.06, 0.44 ± 0.07, 0.32 ± 0.06 *vs* 3.27 ± 0.49, respectively) was significantly suppressed by Fru in LPS-induced RAW264.7 cells (Figures 3A,B). Meanwhile, the upregulation of LPS induced the expression of NF-κB (p50) (1.29 ± 0.13, 1.08 ± 0.10, 0.74 ± 0.07 *vs* 2.28 ± 0.35, respectively) in the nucleus and was remarkably inhibited by Fru in RAW264.7 cells (Figures 3C,D).

### Effects of Fru on MAPKs Signaling Pathway in LPS Stimulated RAW264.7 Cells

**FIGURE 4:**
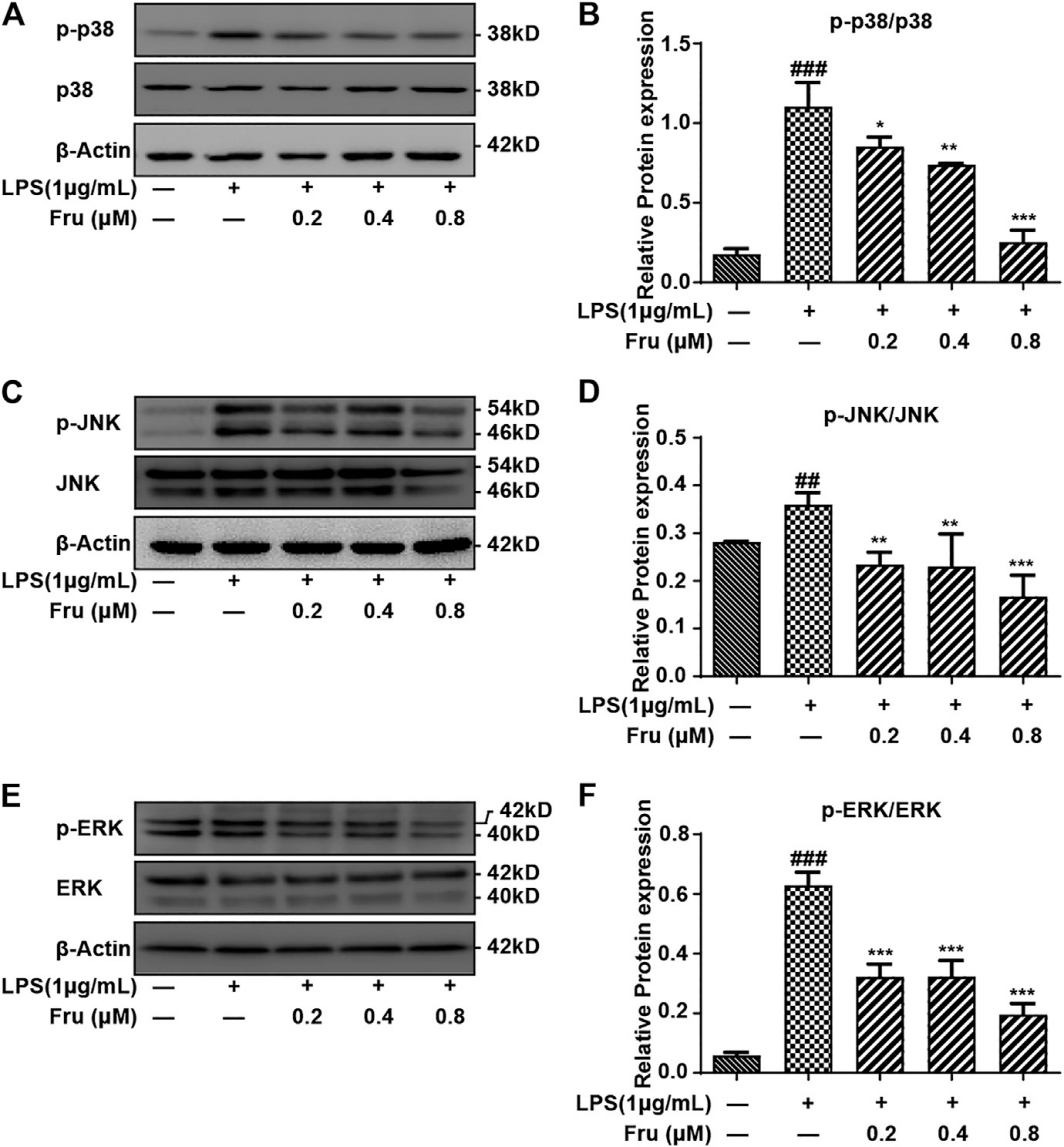
Effect of Fru on the activation of the MAPKs pathway in LPS-induced RAW264.7 cells. The RAW264.7 cells were pretreated with different concentrations of Fru (0.2, 0.4, and 0.8 μM) for 2 h before stimulated with 1 μg/ml LPS for 6 h. The protein expression of p-p38/p38 (Panel **(A,B)**), *p*-JNK/JNK (Panel **(C,D)**), and *p*-ERK/ERK (Panel **(E,F)**) was evaluated with Western Blot. β-actin was used as the internal control. Quantitative data were represented as mean ± SD (*n* = 3, ^##^ *p* < 0.01, ^###^ *p* < 0.001 vs. Control; *p < 0.05, **p < 0.01, ***p < 0.001 vs. LPS).

Fru also significantly inhibited the activation of p38 and dose-dependently down-regulated phosphorylated p38 ratio (0.85 ± 0.06, 0.74 ± 0.03, 0.25 ± 0.07 *vs* 1.11 ± 0.15, respectively) (Figures 4A,B). Furthermore, the phosphorylation of JNK was down-regulated by the pretreatment of Fru in LPS-stimulated RAW264.7 cells (0.23 ± 0.03, 0.23 ± 0.07, 0.17 ± 0.04 *vs* 0.36 ± 0.02, respectively), but exhibited no difference in the action of Fru at 0.2 and 0.4 µM (Figures 4C,D). Fru significantly decreased the phosphorylated ERK ratio (0.32 ± 0.04, 0.32 ± 0.05, 0.20 ± 0.03 *vs* 0.63 ± 0.04, respectively) in LPS-stimulated RAW264.7 cells, but there was no dose-dependance inhibition among different groups (Figures 4E,F).

### Effects of Fru on the Expression of Myd88 in LPS Stimulated RAW264.7 Cells

**FIGURE 5:**
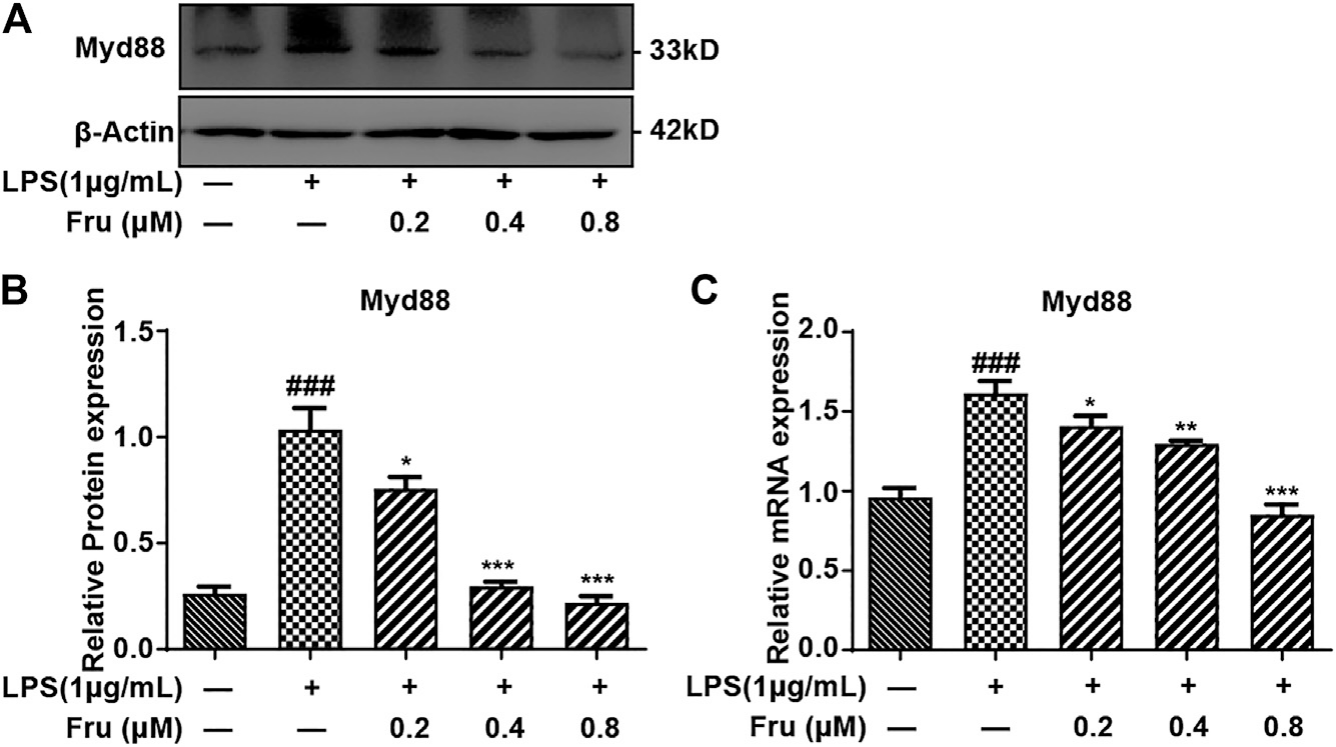
Fru suppressed the expression of Myd88 in LPS-induced RAW264.7 cells. The RAW264.7 cells were pretreated with Fru (0.2, 0.4, and 0.8 μM) for 2 h and then stimulated with or without 1 μg/ml LPS for 8 h **(A, B)** The protein expression of Myd88 was evaluated by Western Blot. β-actin was used as the internal control. **(C)** The mRNA expression of Myd88 was evaluated by qRT-PCR and normalized to GAPDH. Quantitative data was represented as mean ± SD (*n* = 3, ^###^ *p* < 0.001 vs. Control; *p < 0.05, **p < 0.01, ***p < 0.001 vs. LPS).

The qRT-PCR and Western Blot results indicated that Fru remarkably down-regulated the expression of Myd88 in LPS-induced RAW264.7 cells (Figure 5). The relative protein expression of Myd88 in Fru (0.2, 0.4 and 0.8 μM) was 0.76 ± 0.06, 0.30 ± 0.02, and 0.22 ± 0.03, respectively, compared with LPS group (1.04 ± 0.10) (Figures 5A,B). The relative mRNA expression of Myd88 in Fru was 1.41 ± 0.06, 1.30 ± 0.02, and 0.85 ± 0.06, respectively, compared with LPS group (1.61 ± 0.08) (Figure 5C).

### Binding Study of Fru on TLR4-MD2 Complex by Molecular Docking and Cellular Thermal Shift Assay

**FIGURE 6:**
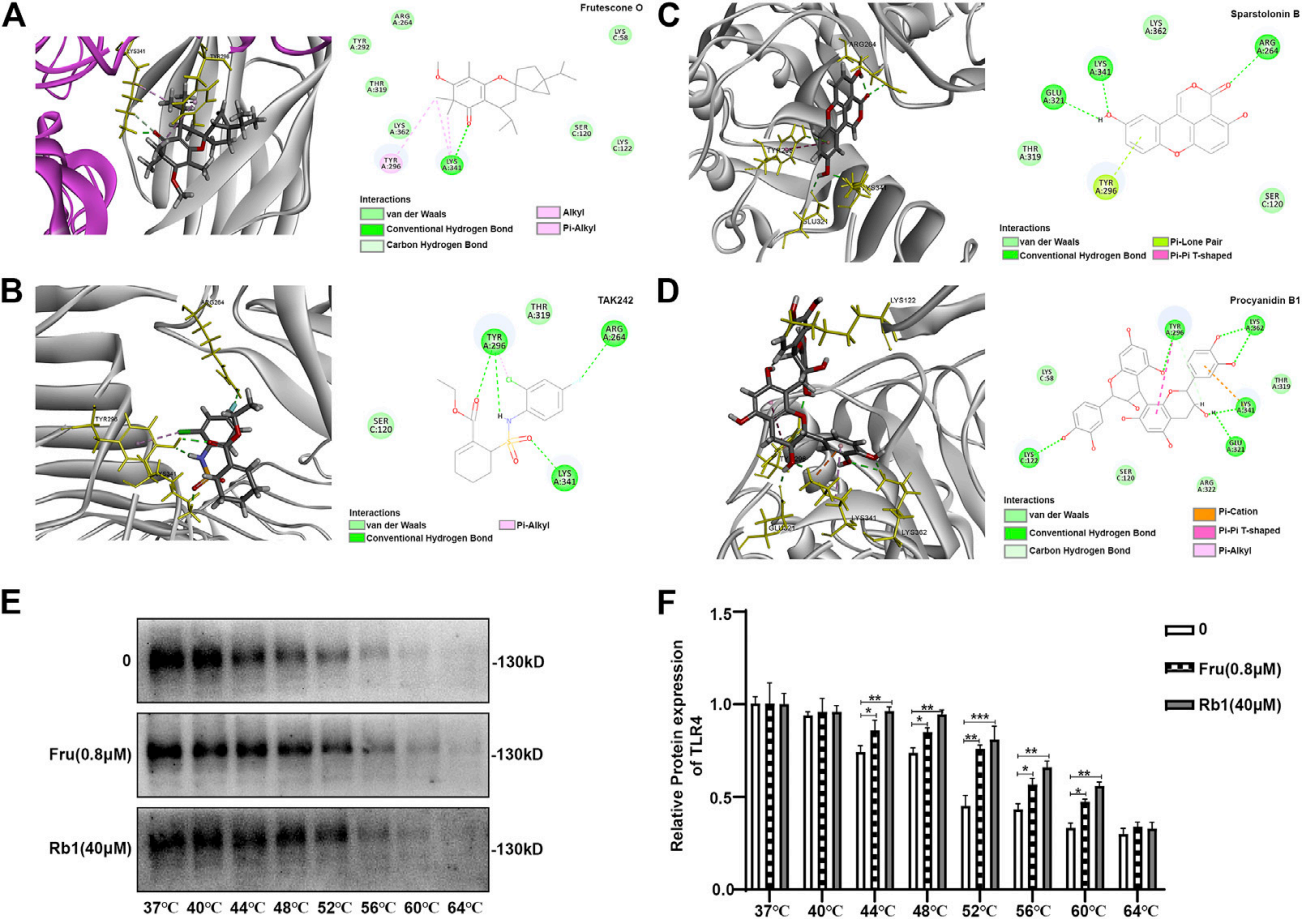
Molecular docking of Frutescone O on TLR4-MD2 complex. **(A)** The close-up view and 2D view of the sites of Fru in the TLR4-MD2 complex. **(B)** The close-up view and 2D view of the sites of TAK242 in the TLR4-MD2 complex. **(C)** The close-up view and 2D view of the sites of Sparstolonin B in the TLR4-MD2 complex. **(D)** The close-up view and 2D view of the sites of Procyanidin B1 in the TLR4-MD2 complex. **(E, F)** The binding study of TLR4 with Fru (0.8 μM) by cellular thermal shift assay. Rb1 (40 μM) served as the positive control and 0 the negative control. Quantitative data was represented as *mean ± SD* (*n* = 3, *p < 0.05, **p < 0.01, ***p < 0.001 vs. negative control).

Fru adopted a favorable conformation at the active site of TLR4-MD2 with an energy of −32.7392 kJ/mol. The carbonyl-oxygen of Fru interacted with amino acids Lys 341 of TLR4-MD2 forming one hydrogen bond (H-bond), bonding distances of 1.85 Å. Fru also could bind to Tyr 296, Lys 341, and Lys 58 of TLR4-MD2 by π-bond (Figure 6A). Meanwhile, TLR4 antagonist TAK242 interacted with amino acids Tyr296, Arg 264, and Lys 341 of TLR4-MD2 at the active site by H-bond and π-bond (Figure 6B). Sparstolonin B, as TLR4 antagonist, also interacted with amino acids Glu 321, Lys 341, and Arg 264 of TLR4-MD2 at the active site by H-bond (Figure 6C). Similarly, TLR4 antagonist Procyanidin B1 interacted with amino acids Lys122, Tyr 296, Lys 362, Lys 341, and Glu 321 of TLR4-MD2 at the active site by H-bond and π-bond (Figure 6D). Furthermore, the thermal stabilization of TLR4 upon binding with Fru was shown in Figures 6E,F. The treatment of Fru (0.8 μM) could increase the TLR4 expression than the untreated group at 44, 48, 52, 56, and 60°C, respectively. Rb1, which could bind to TLR4 and increased the thermal stabilization, was served as a positive control here. These results indicated that Fru could bind to the TLR4 receptor and participated in a powerful and stable interaction with the active site of TLR4-MD2.

## Discussion

Fru, an anti-inflammatory meroterpenoid from *Baeckea frutescens,* demonstrated anti-inflammatory activity by blocking the NF-κB signaling pathway *via* the suppression of p65 nuclear translocation (Hou et al., 2017). In the present study, the anti-inflammatory target and inhibitory mechanism of Fru were further studied in LPS-stimulated RAW264.7 macrophages. Fru suppressed the expression of iNOS and inhibited the production of NO and dramatically blocked the transcription of inflammatory cytokines such as IL-1β, IL-6, and TNF-α. NO is synthesized with the help of iNOS that converts arginine into citrulline producing NO in the process. Furthermore, it involves immune responses by cytokine-activated macrophages, which release NO in high concentrations. NO is involved in the pathogenesis of inflammatory disorders of the joints, gut, and lungs. Therefore, NO inhibitors represent an important therapeutic advance in the management of inflammatory diseases (Sharma et al., 2007).

Fru was found to inhibit the TLR4-mediated Myd88/NF-κB and MAPK signaling pathways and significantly suppressed the LPS-induced upregulation of p50 and *p*-IκBα. After LPS stimulation, the expression of TLR4-related signaling proteins was upregulated in macrophages (Xia et al., 2012; Li et al., 2017; Muralidharan et al., 2018). Myd88-dependent TLRs are important mediators of chronic inflammation in local and systemic inflammation diseases (Kiripolsky et al., 2019). The adaptor protein Myd88 played a significant role in activating the NF-κB signaling pathway and MAPKs signaling pathway (Aderem and Ulevitch, 2000; Kagan and Medzhitov, 2006). In the present study, the molecular docking and cellular thermal shift assay results indicated that Fru could bind to TLR4-MD2 and significantly downregulate the expression of Myd88. Furthermore, Fru inhibited the nuclear translocation of p65, p50 by blocking the phosphorylation and degradation of IκBα.

The pro-inflammatory cytokines, including IL-1β, IL-6, and TNF-α, play an important role in the regulation of inflammation, homeostasis, and immune response (Kwilasz et al., 2015; Raphael et al., 2015; Voiriot et al., 2017). Interestingly, the NF-κB activation is regarded as an important mechanism for TLR4-mediated inflammation in macrophages (Li and Verma, 2002; Simmonds and Foxwell, 2008; Kawai and Akira, 2010; Peirong et al., 2012; Schuliga, 2015). In the NF-κB signaling pathway, the translocation of p50 and p65 into the nucleus was a pivotal step for NF-κB signaling activation (Viatour et al., 2005). It was IKKs-mediated phosphorylation and degradation of IκBα that played an essential role in activating NF-κB signaling (Schuliga, 2015).

MAPKs including ERK, p38, JNK subfamilies, also played an integral role in the signal transduction pathways involved in inflammatory macrophages (De et al., 2005; Kim and Choi, 2010; Kong et al., 2013). Fru strongly inhibited the phosphorylation ratio of p38, JNK, and ERK by suppressing the MAPK pathway transduction. Moreover, BF-ext, the fraction from which Fru was isolated, showed anti-inflammatory activity on xylene-induced ear edema and egg white-induced paw edema in mice (Data are shown in supporting material). These results indicate Fru might serve as a dual inhibitor of NO and PGE_2_. BF-ext also showed therapeutic effects on the phenol-induced cervicitis mice model (Data are shown in supporting material).

In summary, Fru demonstrated an anti-inflammatory function by inhibiting both the NF-κB and MAPK signaling pathways in LPS induced RAW264.7 macrophages. Meanwhile, it also blocked the TLR4 mediated signal transduction by downregulating the Myd88 and iNOS expression. These results indicate that Fru should be the subject of further studies as an anti-inflammatory drug candidate in the future.

## Data Availability

The raw data supporting the conclusions of this article will be made available by the authors, without undue reservation.

## Abbreviations:

Fru, Frutescone O; CETSA, cellular thermal shift assay; IL-1β, interleukin-1β; IL-6, interleukin-6; IκBα, inhibitory κBα; iNOS, inducible nitric oxide synthase; LPS, Lipopolysaccharide; MAPKs, mitogen-activated protein kinases; NF-κB, nuclear factor-κB; NO, nitric oxide; Rb, Ginsenoside Rb1; TNF-α, tumor necrosis factor-alpha; NSAIDs, Non-steroidal anti-inflammatory drugs.
