# Supplementary material for: Frutescone O from Baeckea frutescens Blocked TLR4-Mediated Myd88/NF-κB and MAPK Signaling Pathways in LPS Induced RAW264.7 Macrophages
Source: Front Pharmacol. 2021 Apr 27;12:643188. doi: 10.3389/fphar.2021.643188 (PMC8112673; doi:10.3389/fphar.2021.643188)
Supplement: Supplementary file 2 [file DataSheet2.PDF]

Reagents

Aspirin enteric-coated tablets were purchased from Qingdao Huanghai Pharmaceutical Co., Ltd., specification: 25 mg/tablet, approval number: H37023121. Fuke Qianjin Tablets (QJP), purchased from Zhuzhou Qianjin Pharmaceutical Co., Ltd., specification: 300 mg/tablet, approval number: Z43020027.

Experimental animal

SPF ICR mice, 30 male and 20 female mice, weighing 18-22 g, purchased from Qinglongshan Animal Breeding Farm, Jiangning District, Nanjing City, experimental license number: SCXK (Su) 2017-0001.

Method

Xylene-induced ear edema in mice

Each mouse was intragastrically given a dose of BF-ext (100 mg/kg) or aspirin (200 mg/kg) once daily and for 5 days. At the last day, 1 h after intra-gastric administration, the induction of ear edema by topical application of 0.03 ml xylene on both surfaces of the right ear. The left ear saved as a control. Mice were sacrificed 40 minutes after xylene application. Ear disks of 9.0mm in diameter were punched out. The mass was weighed with an analytical balance to calculate the degree of swelling. Percent ear edema rate was calculated according to the following formula :

Edema Rate (%) = $\frac{W_r - W_l}{W_l} \times 100\%$ (W_r : disk of the right ear; W_l : disk form the left ear)

Egg white -induced paw edema in mice

Once daily, for 5 days the rats were given BF-ext and aspirin orally at a dose of 100 mg/kg by feeding tube. Measurement index and calculation method: 1 hour after the last administration, egg white was subcutaneously injected into the left toe of the mouse, and the swelling value of the left and right ankle of the mouse was measured 60 min with a vernier caliper. The right foot was used as a control to calculate the degree of swelling (The thickness difference between the left and right feet of the mouse) and the rate of swelling.

Phenol induced cervicitis model in mice

20 female ICR mice were randomly divided into control group, model group, QJP (1g/kg)

group, and BFext (100 mg/kg) group, n = 5. A 20 μL of phenol glue was injected in vagina, once a day, for 3 consecutive days. And the mouse was found with red and swollen vaginal lipstick and pus secretion. 1 hour after the last administration, the blood sample was collected by eyeball method. After 2 hours, blood samples were centrifuged at 3000 g for 5 minutes, and the supernatant was collected for ELISA analysis. The content of IL-6 was directly analyzed by ELISA according to the manufacturer's instructions. The whole uterus of mice was dissected and separated, and the cervix was immersed in 10% formaldehyde for 48 h and stained with hematoxylin-eosin (HE) for histopathological analysis.

Results

Xylene-induced ear edema and Egg white -induced paw edema in mice

Compared with the control group, the ear swelling degree and swelling rate of mice in the BFext (100mg/kg) group were significantly reduced, the swelling degree was decreased from 6.97 mg to 3.50 mg, and the swelling rate was decreased from 141.54% to 67.10% (showed in Table 1 and Fig.1). The ear swelling degree of mice in the aspirin (200 mg/kg) group was reduced to 3.43 mg, the swelling rate was reduced to 66.19%. Compared with mice in the control group, the degree of swelling in BFext group was decreased from 0.87 mm to 0.32 mm, and the rate of swelling was decreased from 36.60% to 13.83%. The degree of swelling in aspirin group was decreased from 0.87 mm to 0.36 mm, and the rate of swelling was decreased from 36.60% to 15.10% (showed in Table 2 and Fig.1). These results indicated that BFext exhibit anti-inflammatory effect *in vivo*.

Table 1 The effect of BFext on xylene-induced ear swelling in mice（n=5，Mean±SD）

Groups	n	Dose	Degree of swelling (mg)	Rate of swelling (%)
Control group	5	—	6.97 ± 0.80	141.54 ± 10.87
Aspirin group	5	200 mg/kg	3.43 ± 0.29***	66.19 ± 6.50***
BFext group	5	100 mg/kg	3.50 ± 0.45***	67.10 ± 9.20***

Note: *** p < 0.001 *vs*. Control group

Table 2 The effect of BF-ext on mouse foot swelling induced by egg white (n=5，Mean±SD)

Groups	n	Dose	Degree of swelling (mm)	Rate of swelling (%)

Control group	5	—	0.87 ± 0.09	36.60 ± 4.11
Aspirin group	5	200 mg/kg	0.36 ± 0.12***	15.10 ± 4.80***
BFext group	5	100 mg/kg	0.32 ± 0.07***	13.83 ± 3.13***

Note: *** p < 0.001 *vs*. Control group

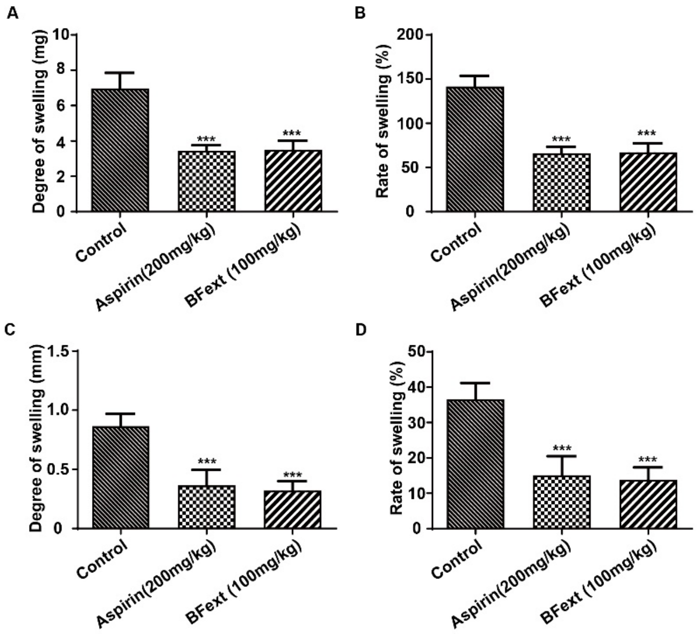

Fig.1 The anti-inflammatory effect of BFext *in vivo*. A and B) The effect of BFext on xylene-induced ear swelling in mice. C and D) The effect of BFext on mouse foot swelling induced by egg white. (n = 5, *** p < 0.001 *vs*. Control group)

Phenol induced cervicitis model

The uterus of mice in the model group showed the infiltration of a large number of inflammatory cells in compared with control group. And the inflammatory symptoms of the QJP group and the BFext group were significantly improved compared with the model group (Fig.2A). The IL-6 levels in the serum of the model group were significantly increased compared with the control group. However, the IL-6 levels in the serum of the BFext and QJP groups were decreased after treatment (Fig.2B). These results indicated that BFext had protective effect in cervicitis mice.

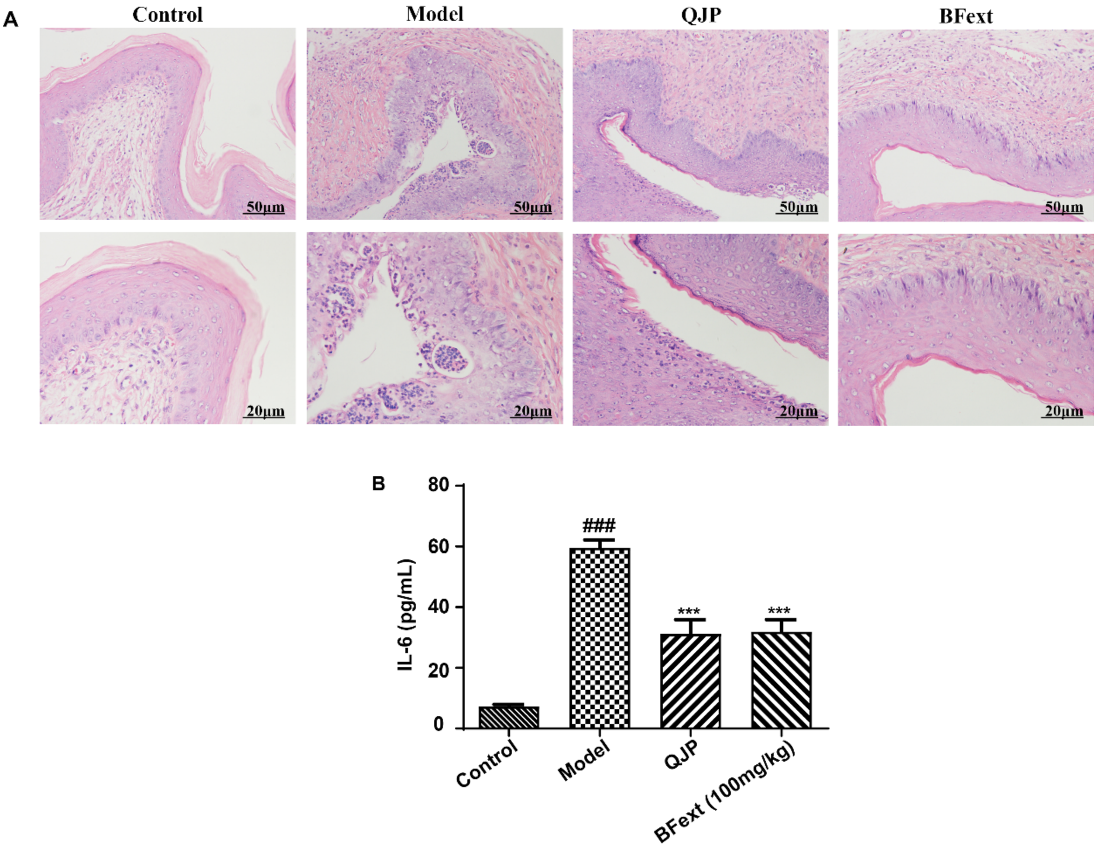

Fig.2 The effect of BFext on cervicitis in mice induced by phenol glue *in vivo*. A) The HE results of BFext in cervicitis mice. B) The effect of BFext on the IL-6 levels in the serum in cervicitis mice. (n = 5, ### $p < 0.001$ *vs*. Control group; *** $p < 0.001$ *vs*. Model group)
